# Black feminism and Artificial Intelligence: the possibilities and limitations of contesting discriminatory AI from a critical social theory perspective

**DOI:** 10.3389/fsoc.2025.1602947

**Published:** 2025-07-23

**Authors:** Laura Schelenz

**Affiliations:** International Center for Ethics in the Sciences and Humanities (IZEW), University of Tübingen, Tübingen, Germany

**Keywords:** Black feminism, Artificial Intelligence, diversity, critical theory, technology development

## Abstract

Artificial Intelligence (AI) has been challenged in recent years by critical scholars inspired by American Black feminism and questions around diversity, inclusion, and justice in and through AI systems. This paper takes a closer look at the application of Black feminism as a critical social theory, which originated as a theory protesting the oppression of Black women as a group in the United States. The paper reveals the benefits and limitations of the theory in contesting AI-based sorting, classification, personalization, recommendation, and population-based predictions of different groups of technology stakeholders. Through conceptual analysis and reference to recent use cases of AI applications, the paper showcases the promise of an intersectional-type analysis and a structural perspective enabling the in-depth analysis of technology stakeholders' experiences with AI. The paper also highlights limitations of the theory in contesting AI, which inspires a discussion of constraints on critical scholarship on AI more broadly. The papers' findings and arguments are relevant to those engaging in critical conceptual, qualitative research on the design and implications of AI-based computer systems.

## 1 Introduction

Artificial Intelligence (AI) has been subject to critical evaluation in computer science (see the field of Machine Learning Fairness, Oneto and Chiappa, 2020), but also in science and technology studies (STS), philosophy, ethics, sociology, and the humanities. AI has become an integral part of everyday life covering all aspects of society at global scale: AI may include prediction and decision-making tools as well as (generative) assistant technologies in education, hiring and recruitment, criminal justice, communication, personal (intimate) relationships, medicine, health, science, climate and all areas of technology development (Hacker, 2025). Given the permeation of AI in society in combination with concerns about bias in and through AI, critical scholarship on AI has gained traction in the humanities and social sciences (Hanemaayer, 2022). Particularly powerful critiques of AI include scholarly works inspired by US-American Black feminism (Schelenz, 2022a), a theory and practice developed by Black women in the United States to theorize and protest the oppression of Black women at the intersection of race, gender, and class (Collins, 2000a). This paper discusses an American Black feminist approach to contesting AI. The approach is interesting because it adds a structural, power-centric perspective to the debate around AI ethics. While other critical theories such as Marxism, decolonial studies or queer studies similarly consider power relations in society and technology, Black feminism is interesting because it enables an intersectional-type analysis that foreground AI's impact on technology stakeholders at the intersection of race, gender, class, sexuality, ability, and more. While a Black feminist intersectional-type approach to AI is certainly not the only feminist response to AI (see the wealth of feminist approaches in Browne et al., 2023), Black feminism is distinct because it builds on the structural experiences of Black women.

Black feminisms exist in plurality and have emerged in different regions in Africa, Latin America, the Caribbean, and Europe (Emejulu and Sobande, 2019; Norwood, 2013; Rodriguez et al., 2016). This paper focuses on US-centric Black feminism because US-based Black feminist works have increasingly targeted technology design and development as a site of critical inquiry (I will come back to that in a moment). US-American Black feminism combines theory and practice of resistance to the oppression of Black women. Intersectionality is an important analytical tool developed by Black feminists to reveal intersecting experiences of marginalization (Cooper, 2016). The work that Black feminists do is grounded in a normative concern for social justice and has driven Black women's activism around social change since U.S. slavery (Guy-Sheftall, 1996).

Research inspired by Black feminism (in the following abbreviated as BF) has challenged AI-enabled technologies in recent years. This includes critiques of algorithmic bias and discrimination (Buolamwini and Gebru, 2018; Buolamwini, 2019a; Howard and Borenstein, 2018; Noble, 2018; Hampton, 2021), critiques of color-blindness in the technology industry (Daniels, 2015), accounts of racism against Black women in computer science (Thomas et al., 2018), studies of race as a system of power and its entanglement with technology (Benjamin, 2019b), frameworks for the design of technology (Costanza-Chock, 2020; Erete et al., 2023, 2018; Kumar and Karusala, 2019), and frameworks for data collection and analysis (D'Ignazio and Klein, 2020; Marshall, 2023). BF has become an important foundation of the critical study and design of society and technology, to the extent that the concepts and ideas of BF are also employed by White women and queer or transgender scholars.^1^

Given the increased popularity of BF in the critical study of AI-enabled technology, it is important to take a closer look at the potential and limitations of the theory. This paper therefore answers the following research questions: What tools of analysis and critical thinking can the theory and practice of Black feminism offer for the study and design of AI? What are limitations of Black feminism as a critical social theory in contesting AI? The paper focuses on the contestation of AI-based technologies that specifically work with concepts of diversity in their design (e.g., in the construction of datasets or features of an application) or that consider and cater to diverse groups of technology stakeholders. The focus on diversity and AI as the overarching use case was chosen as recent years saw not only an uptake in Black feminist critique but also more awareness of the lack of diversity in design (Zou and Schiebinger, 2018; Erete et al., 2023).

Following a conceptual analysis of BF writings and their application to STS, the paper argues that US-American Black feminism as a critical social theory offers a rich foundation for contesting AI-based technologies that are harmful to, e.g., Black women. BF is particularly suited to critically evaluating diversity-related matters of AI, among other reasons because BF has a long tradition of analyzing notions of societal difference, which increasingly inform the design of automated personalization, recommendation, adaption, and population-based predictions (e.g., intersectional-type analysis, Crenshaw, 1989; Noble, 2016). Aside from arguing for the potential of contesting AI with BF, the paper also addresses its constraints. BF shows some limitations in critically studying AI: first, while BF focuses successfully on power systems, these are mostly limited to gender, race, and class. Second, while BF-inspired analyses are excellent at making visible the lived experiences of technology stakeholders (e.g., those subjected to algorithmic mediation), the way that those experiences are present may inadvertently essentialize them.

The third limitation points to a tension in BF about using established language and methods (including problematic constructs like “man” and “woman”) vs. refusing them and creating new ones in an analysis of AI bias. The last limitation shows overall constraints on critique from a critical social theory perspective, as the critique often comes from within the power systems which it tries to challenge.

The findings of the paper inspire a discussion of constraints on the critical study and design of AI more broadly. As stated above, BF aspires to create social change because Black women's consciousness of oppression animates their call for social justice (Collins, 2000a, p. 3). Yet when transformation toward socially just AI frameworks seems unreachable, because most critique operates from within rigid power structures that make it difficult to escape established ways of thinking and doing, where does this leave critical scholarship? In the discussion, I argue in favor of a continuum of action on AI, including structural critique that aims at transforming existing AI frameworks as well as smaller, strategic steps toward betterment.

## 2 Materials and methods

This paper contributes to the debate around contesting harmful AI applications and effects in relation to questions of diversity by discussing the potential and limitations of BF theory as a vantage point for critique. Focusing particularly on questions of diversity means that the analysis of the theory's potential and limitations considers arguments and use cases that are related to diversity. Some use cases chosen for the analysis fall into the broader category of diversity-aware or diversity-sensitive technology. To briefly define what I mean by diversity and diversity-awareness, diversity refers to a conceptual idea of difference (e.g., among users of technology) or a normative claim toward fairness, inclusion, and justice, (e.g., of different stakeholders in the digital society, Schelenz et al., 2019). Diversity-aware technology is a technology that, in its design, draws on a concept of difference and/or a normative claim related to inclusion, representation, equality, and more (Schelenz, 2023a).

The focus on diversity-related arguments and use cases was chosen because more and more computer scientists and technology designers are interested in diversity. For instance, diversity is increasingly embedded as a concept and/or a value into algorithmic models, data collection processes, and the design of user interfaces: In human-computer interaction, diversity concepts of the user are employed to make the design of a system more responsive to the diverse needs of users (Himmelsbach et al., 2019). In AI-based recommendation systems like Netflix or Amazon, different items (products or content) to be recommended to the user are perceived as diverse, plus designers diversify recommendations to achieve fairness (Sonboli et al., 2020; Ekstrand et al., 2018) and user satisfaction (McNee et al., 2006). Furthermore, diversity is considered in balanced datasets that include information about different populations to counter data and algorithmic bias (cf. Buolamwini and Gebru, 2018; Kärkkäinen and Joo, 2019). With increased interest of designers in diversity, I observed in prior work that the way that diversity is leveraged in the technology industry often constitutes oversimplified, binary, and naturalizing operationalizations of (human) difference (Schelenz, 2022b). This is where a critique inspired by Black feminism comes in, and the potential and limitations of this critique are evaluated in the paper's analysis.

The analysis is structured as follows: First, I conducted a conceptual analysis of the key features and arguments of BF as well as its potential for producing comprehensive critiques of contemporary socio-technical practices. The conceptual analysis was conducted by reviewing writings on the theory and method of BF. The literature used for this exercise is reflected in a classic canon of Black feminist writings, represented in the anthology by Guy-Sheftall (1996). I thus relied on an older, classic body of literature represented by the likes of Kimberlé Crenshaw, Patricia Hill Collins, bell hooks, and Audré Lorde. More recent post-structuralist and queer Black feminist literature including by Jennifer Nash and Jasbir Puar was added to gain a full view of the theory's strands and evolution. The literature addressing specifically technology and AI using Black feminist argumentation includes among others Benjamin (2019a,b), Noble (2018, 2016), Marshall (2023), Buolamwini (2023).

Second, BF tools and arguments were applied to different cases or examples of AI and diversity in order to identify the benefits and limitations of the theory. For instance, one use case is the study of algorithmic bias and discrimination in the field of AI ethics, which conducts research into how social groups are treated differently through algorithmic mediation. In recent years, ethicists have pointed to numerous cases of profiling, misrepresentation, erasure and disparate treatment through AI, affecting core areas of life including education, job recruitment and hiring, and criminal justice (Heesen et al., 2021; Fabris et al., 2025). I have evaluated how BF adds to AI ethics or complements arguments of algorithmic discrimination. By way of example, my analysis determined that BF primarily promotes a structural perspective on societal relations rather than the focus on individual behavior or identity, which means that AI bias can be scrutinized from a BF perspective in a way that considers the broader social fabric. BF then allows AI-based applications to be assessed as to their impact on power relations: AI may alter and transform or reinforce and cement long-held gender and racial relations as well as injustices in society.

Another use case is the design and evaluation of AI-based technology that seeks to foster the social inclusion of migrants and refugees. In this context, diversity relates to social inclusion in the sense that migrants face different challenges than the majority society, e.g., with regard to language and access to computer systems. My own previous research has looked into how an (AI-based) chatbot can support Afghan refugee women in settling in Germany (Schelenz, 2023a). In the course of this work, I tested a prototype of the chatbot with Afghan refugee women and conducted a focus group with participants (Schelenz, 2023b). This use case was chosen to evaluate how Black feminist arguments and approaches fare in a context outside the USA, because it helps make a claim about the applicability of the theory to technology-related use cases across regions. More examples for diversity-related technology development are included along the text to sharpen arguments and help the reader understand the particularities of a Black feminist critique of AI.

Much of the paper involves my own interpretation and judgment of how Black feminist arguments can enrich discussions and debate contesting AI. While my arguments are of course grounded in a rich foundation of literature (both in the field of Black feminist thinking but also the critical study of technology inspired by Black feminism), investigating, testing, and evaluating a theory remains a highly qualitative and at times subjective task of interpretation. I hope that scholars will critically engage with the soundness and substance of the arguments made in this paper and perhaps relate them to other use cases of AI or investigate a different critical theory (whether it is decolonial studies, queer studies, etc.) as to its potential and limitations in contesting AI.

## 3 Results

### 3.1 Part A: the benefits of Black feminism in contesting Artificial Intelligence

The results section first presents the benefits of BF in contesting AI.

#### 3.1.1 Benefit 1: a structural perspective offers a multi-dimensional critical analysis of AI

An important feature of BF is its structural perspective on society and technology, which is inspired by an intersectional-type analysis. Intersectionality is one of the core products of Black feminist thought and moved into mainstream feminist politics around the 2000s (Yuval-Davis, 2006, p. 194). Established as a term by Crenshaw (1989, 1991), intersectionality reveals interlocking forms of oppression when systems of power such as race and gender interact (also see Collins, 2000a, p. 227; Collins and Bilge, 2016). Power systems are conglomerates of knowledge, narratives, and symbols that materialize in the creation and operation of institutions, policies, and practices. Power systems rely on the social construction of human difference. They structure society and technology by advancing certain norms and othering those people and practices that fall outside the norm.

Power systems then become systems of oppression for those who are deemed “Other” to the norm (Collins, 2000a, p. 4). To make explicit the hidden norms produced by power systems, BF considers the social groups that are structurally advantaged and disadvantaged in society: the norm of race is White, of gender is male, of class is middle or upper class, of sexuality is heterosexual, of ability is abled-bodied.^2^ Black women as a group are structurally disadvantaged in society and technology as they are consistently “othered,” whether as people or technology users (Noble, 2018) or designers (Twine, 2018). A structural perspective thus reveals the experiences of different groups (or “structural identities”) in society.

The emphasis of a structural perspective in BF should not dismiss individual and personal influences on stakeholders' experiences with technology. However, an individual perspective often renders invisible that individuals are influenced by larger structures that shape their individual behavior (Collins, 2000a, p. 171). For example, racism is not merely an individual attitude but promoted through institutions that affect individuals (e.g., schools, administrations, etc. Collins, 2000a, p. 227; Feagin, 2006). Race as a system of power is produced and reproduced via institutions and the collective of individuals who are socialized in said institutions to internalize a series of “knowledge” (e.g., race science, Rusert, 2017; Subramaniam, 2014), narratives (of who is considered the norm, Reddy, 1998), and symbols (skin color, bodily appearance, hair, Hooks, 2015b) that degrade Black women.

A structural perspective can also help theorize diversity and difference. Black feminists have demonstrated that Black women have structurally different experiences than White women. For example, sexual violence is experienced differently by White and Black women with Black women being turned away at the women's shelter or immigrants not finding support in their native language (Crenshaw, 1995). This is because of the co-constitution of systems of power and the intersecting oppression that Black women face (for a history of Black feminist intersectional thinking, see Brah and Phoenix, 2004). Structural difference or structural diversity is then tied to diverging experiences of oppression (and privilege, Collins, 2000a, p. 25f). The idea of structural diversity as the different experiences of groups is beneficial because it helps researchers and designers understand how AI-based technology affects people differently depending on their socio-economic-political standing in society.

In application to the study of AI-based technology, BF has the capacity to shed light on the lived experiences of technology stakeholders as they are shaped by power systems (Schelenz, 2022a). BF can reveal a technology stakeholder's position at the intersection of power systems such as gender, race, and class, and how this position materializes in specific types of oppression. For instance, Black women are impacted by design systems and institutions that have been found to exhibit bias against Black women due to an overrepresentation of White male perspectives in datasets and design teams (Criado Perez, 2019; Wachter-Boettcher, 2017, p. 20). Biased and derogatory cultural representations of Black women as “Mammies,” “Jezebels,” and “Sapphires” are disseminated at scale in AI-optimized search engines and chat bots, which act as knowledge gatekeepers and cement harmful stereotypes (Salinas et al., 2024, p. 24ff; Noble, 2018, p. 98).

Black women may further experience economic injustice as AI-based job applicant rankings can disadvantage Black women because of a combination of gender and racial biases: Hannák et al. (2017) show that users who are read as Black and female are ranked lower than candidates from other social groups including White women. As a consequence, Black female applicants do not appear on the radar of employers or they are seen as less qualified. This bias originates in ratings and reviews, as written feedback contains fewer and less positive adjectives for Black women than for other groups (Hannák et al., 2017). With the help of a structural perspective, these experiences of political, cultural, and economic marginalization entangled with the use of AI can be made visible and related to larger social and historical contexts.

Apart from hiring and recruitment, another prominent topic in AI ethics that foregrounds questions of diversity is the visual representation of humanoid AI systems. Cave and Dihal (2020) have argued that imaginations of AI in visual productions are overwhelmingly racialized as White. Humanoid robots but also chatbots carry physical features that resemble a White face, and voice assistants use middle- class “White” American English instead of African-American Vernacular English. Black feminist analyses point to the not so subtle messaging behind these interpretations of AI identity. Benjamin (2019b, p. 57) discusses an advertisement from the 1960s which shows a robot as a dehumanized servant, whereas the text reads: “Slavery will be back” and “Slavery will be here to stay.” In the past leading to the present, imaginations of robots by White people often involved a desire to dehumanize and command inferior “beings” (Benjamin, 2019b, p. 56). This resembles White attitudes during slavery, when Black women were forced to work not only in the cotton fields but also as servants in the household (Hooks, 2015a, p. 24ff). Going a step further, the Whiteness of robots may also be an intentional move toward an imagined White utopia, in which Black women as domestic workers are entirely removed from the White family's home (Cave and Dihal, 2020, p. 94; Rhee, 2018, p. 94). A critical Black feminist analysis helps reveal those links and understand historically-grown dynamics of oppression which become entangled and embedded in the design of AI systems.

#### 3.1.2 Benefit 2: Black feminist tools of analysis can be applied across regional and cultural contexts

Since power systems follow a similar logic (of creating a norm and an “Other”), Black feminism helps to theorize social difference across geographic and cultural contexts. Black feminists have stressed that systems of power are universal to the extent that they shape the experiences of Black women and women of color in all countries and cultures. There is an important clarification to be made here. The argument is not that experiences of marginalization are the same across geographic and cultural contexts. Rather, the systems of power (race, gender, class) shaping those experiences follow a similar logic in that they establish a norm and materialize as barriers for women's self-actualization (Dhamoon, 2015, p. 26ff).

The benefits of attending to local particularities is that power relations can be considered with regard to their materialization in a specific location. For instance, racism in Europe works differently than in the USA. It is usually expressed as cultural racism (especially against Muslim immigrants, Chin, 2017) and triggered by cultural, religious or ethnic symbols such as the hijab. Despite multicultural narratives, “white cultures are still posited as superior to other cultures and are thus normative whereas non-white cultures are doomed deviant and inferior” (Salem and Thompson, 2016, p. 13). In application to diversity and AI-based technology, this means that AI design must pay attention to how power materializes at the local level.

The case of a mobile phone application for Afghan refugee women in Germany illustrates the benefits of a BF approach (Schelenz, 2023b). According to the study results, Afghan women experience insecurity in navigating their new environment in Germany, e.g., fear of getting lost and missing an appointment at the immigration office. Initially, the experience of uncertainty in a new environment seems universal, affecting well-situated travelers and refugees alike. Yet power systems (particularly gender, ethnicity, language, nation/state) produce a specific experience: due to cultural racism in Germany, Afghan women may be hesitant to approach people in the street for help in navigating the city.

An app may then be a welcome support system but requires real-time, on-the-go assistance from volunteers or peers, simple navigation, and audio communication, according to study participants (Schelenz, 2023b, p. 18). An obstacle is that technology is often offered in English and uses the Latin alphabet, both mostly foreign to Afghan refugee women (Sabie and Ahmed, 2019, p. 223). Afghan refugee women may learn German in Germany, the German government does not offer free language classes to Afghan refugees as they do to, e.g., Syrian refugees (Weibert et al., 2019, p. 4). The modalities of interaction with AI-assisted technology are thus influenced by geopolitically situated power dynamics. BF helps reveal those dynamics and contest the broader policy shift toward digital, AI-based services by the German migration management system, which are difficult to manage for many refugees.

### 3.2 Part B: limitations of Black feminism in contesting Artificial Intelligence

Despite the great potential of BF for the study and design of AI-based technology, especially in relation to questions of diversity and inclusion, there are also limitations.

#### 3.2.1 Limitation 1: BF pays little attention to power systems like language, religion, and wealth but they are important elements of a critical analysis of AI

While Black feminism is an excellent theory to make sense of the work of power, how power relations come about, and how power systems interact, there is unequal attention to different power systems. Nash (2008, p. 9) criticizes the hegemony of gender and race in Black feminism:

“Crenshaw's seminal analysis of the ways in which black women's experiences of sexual assault and domestic violence are mediated by both race and gender neglects the ways in which these experiences are also complicated by class, nationality, language, ethnicity, and sexuality.”

Collins (2015, p. 2) suggests that the following systems of power are dominating intersectional analyses: “race, class, gender, sexuality, ethnicity, nation, ability, and age.” Lorde (1980) suggests there is an overemphasis on gender and suggests to include “race, sexual preference, class, and age” in analyzing structural difference. Yuval-Davis (2006, p. 202) cites a presentation by Helma Lutz who establishes 14 systems that structure social difference: “gender; sexuality; ‘race'/skin-color; ethnicity; nation/state; class; culture; ability; age; sedentariness/origin; wealth; North–South; religion; stage of social development.” The expansive view of Lutz points to the existence of power systems that have received little attention in Black feminism and may be more visible in post-colonial and decolonial feminisms that place emphasis on women's experience in the Global South, as immigrants, and transnational women (Dhamoon, 2015, p. 27; Khan, 2018; Mirza, 2009, p. 7).

In the use case of the mobile phone app for Afghan refugee women, language and digital literacy play a significant role (Schelenz, 2023b, p. 17). The use case showcases how (socio-technical) power relations are created through “linguistic ideologies, language policies, […] and communicative practices” (Windle et al., 2020, p. 11). The dominance of the English language has shaped the design of technology from the start. Warschauer (2004, p. 203) observes that the American Standard Code for Information Interchange based on the English and Roman languages was used in the initial stages of the development of the Internet. Engineers outside of the Western context were thus unable to participate in shaping the Internet, giving American and European designers a head start before the world standard for text was switched to Unicode (Warschauer, 2004, p. 203).

On a different level, workers in call centers in the Global South that provide technology support to American customers are encouraged to fake a White English sounding name and accent to appear White to the customer (Poster, 2019, p. 152). Not only is English the dominant language in the global digital economy, but White middle-class English is preferred over African-American Vernacular English (also in voice assistants and chatbots, Cave and Dihal, 2020, p. 690; Windle et al., 2020, p. 15). BF traditionally exhibits a blind spot with regard to language as a structure shaping power relations. This becomes a challenge for contesting AI, its economic context and impact on low-literate and non-English-speaking populations. In light of the increased use of large language models, language (bias) may shape the future of digital search, information, and communication (Bender et al., 2021).

Religion is another system of power that has received less attention in Black feminism and intersectional analysis than race and gender. As Weber (2015, p. 23) observes, “discussions of intersectionality have been hesitant to engage faith and religion, other than to occasionally list religion as one in a list of relevant differences.” Yet religion as a system structures social relations and creates racialized and gendered hierarchies through “organized institutions that produce particular norms and forms of belonging” and exclusion (Weber, 2015, p. 23). The co-constitution of race and religion is traced by Robinson (2019), who draws on Black feminist writer Wynter (2015), to the construction of the racialized human subject during modern colonial expansion. The idea of White European superiority is in large parts shaped by the transformation of the “human subject” as governed by god to the “human subject” as governed by the state. In other words, secularization established a notion of the human as subject to the state but also as “homo politicus,” who governs the state. In this shift, man suddenly enacted the role of god: the European man (in particular) gains the status of the savior who is to “maintain the stability, order, and territorial expansion of the state” (Robinson, 2019, p. 259f). Furthermore, the idea of a universal monotheistic religion (as opposed to locally specific traditions, practices, beliefs) is exported with Western imperialism, and the religious norm of (White) Christianity is installed as part of colonization and nation-state formation (Robinson, 2019, p. 259f). To theorize religion as a system of power is not to say that Christianity, Judaism, or Islam are inherently oppressive. Religion, like race and gender, are macro-structures, and the way religious norms have been established, e.g., through male interpretations of religious texts, can be reframed to align with feminist notions (Turman, 2016; Kirk-Duggan, 2014; Day, 2016).

Coming back to the use case of Afghan refugee women's interaction with AI-based technology, religion plays a role. Afghan refugee women report that they have a high priority for privacy and safety when interacting with social media (Schelenz, 2023b, p. 13). This may be motivated by religious gender norms, as interpretations of Islam shape the expectation of women's and men's behavior online as much as offline. Privacy and safety of the woman (and by extension the family) are important concerns for Muslim women (Mustafa et al., 2020, 3). Afghan women are expected not to share photographs and real names (Ahmed et al., 2022) or interact with men outside the family in social media (Shahalimi, 2022, p. 113). Yet these norms are rarely taken into account when designing technology. Instead, hegemonic Christian or secular gender norms dominate technology design. A power-centric analysis can reveal this bias and shift perspective to highlight the need for women-only online spaces, which have been used by Muslim women before (Piela, 2012). Regarding religion through the lens of power can thus be an efficient element in the toolbox to critically evaluate AI systems and their impact.

Finally, wealth as a power system has gained little attention in Black feminism vis à vis gender or race. Wealth or capital is a power system that normalizes the accumulation of wealth through the extraction of human and planetary resources. According to the logic of wealth, the redistribution or minimization of wealth is bad. Kelly (2023) calls this wealth or capital bias. Kelly argues that “wealth supremacy” is entangled with White supremacy as the exploitation of Black and Brown people serves to maximize the wealth of those who own it: White people (Kelly, 2023, p. 12; 41f). However, the way that intersectional-type analysis deals with economic inequalities is through “class,” e.g., pointing to the experiences of multiple burdens of Black working class women compared to White women or Black men (Collins, 2000b). Bender et al. (2013, p. 247) argues that class fails to capture the complex macrostructures of production and domination (“gesellschaftliche Produktions- und Herrschaftsverhältnisse,” Garske, 2013, p. 248) that shape social life.

A focus on wealth or capital can account for dynamics of AI-supported data extraction that seek to maximize financial wealth, a phenomenon that Zuboff (2019) calls “surveillance capitalism.” Surveillance capitalism uses behavioral data to know, predict, and nudge the behavior of people. Instead of producing or selling services or goods (this is a side effect), surveillance capitalism seeks maximum information (Zuboff, 2019, p. 513), and thereby maximizes wealth (advertising based on behavioral data is a source of immense income for the tech industry). Although Zuboff does not relate surveillance capitalism to capitalism's co-constitution with slavery and imperialism, the practice of massive data collection has been suggested to be violent to minorities (by “including” them under the pretext of algorithmic improvement in a harmful system of data exploitation, Hoffmann, 2020) and colonizing (by extracting data from African peoples but advancing sophisticated data protection regimes in Europe, Coleman, 2019).

#### 3.2.2 Limitation 2: BF-inspired analyses may inadvertently essentialize experiences of technology stakeholders, making it difficult to contest AI in a way that gets to the origins of the discontent

As we have seen in the discussion of the benefits of BF, using an intersectional-type structural analysis and perspective can reveal technology stakeholders' particular socio-technical experiences interacting with AI. There is a significant benefit of presenting experiences rather than identities in contesting AI's impact on different groups. Focusing on identities in a social critique, also known as the additive approach of intersectionality, tends to present social identities as naturalized, implying a biological or genetic constitution of the identity group in question (Yuval-Davis, 2006, p. 199). Focusing on experiences is less essentializing in a way. This said, there has been criticism in BF itself of the way that experience may inadvertently have essentializing effects as well. Nash (2008, p. 12) argues that intersectionality's focus on experiences of oppression obscures differences within the totality of Black women's experiences; some women might experience privileges:

“In painting black women, for example, as wholly oppressed and marginalized, intersectional theory can not attend to variations within black women's experiences that afford some black women greater privilege, autonomy, and freedom.”

This becomes relevant in the realm of technology design and development as some Black women may be more privileged than others, e.g., in terms of having access to a device and the Internet. This privilege of access to technology is not self-evident for resource-constrained Black people, refugees, and people of color in some areas of the Global South (Alden, 2003; Khan, 2018). At the same time, Black women in computing—despite the multi-layered discrimination they experience (Rankin et al., 2021)—can be considered privileged in terms of education and economic standing (Liang et al., 2021, p. 28). One risk of presenting experience is thus that it does injustice to a diversity of experiences within a set of experiences.

However, more importantly for contesting AI efficiently, while experiences of privilege and oppression are influenced by power systems, the context that determines experiences (i.e., the exact work of power systems/relations) is often removed from the narration of the experience, which makes the experience appear natural (Perpich, 2010, p. 14). This is problematic because experiences become perceived as facts rather than interpretations of social phenomena that point to underlying power structures, which *can* be changed. Mirza (2009, p. 5) writes:

“Appeals to experience risks obscuring regimes of power by naturalizing some experiences as normative, and others as not, leaving the processes that structure dominance in tact (Scott, 1992). A black and post-colonial feminist standpoint does not valorise experience as an explanation or justification in itself, but should be seen as an interpretation of the social world that needs explaining.”

For a successful critique of problematic AI application and decision-making, the risk of essentializing experience becomes quite significant. Computer scientists who are already sensitive to human-centric and critical frameworks and engage in critical analyses of AI-based technologies increasingly emphasize the importance of accounting for structural experiences of technology stakeholders, e.g., through story-telling (Ogbonnaya-Ogburu et al., 2020), autobiography (Erete et al., 2023) or ethnography (Wong-Villacres et al., 2018). Producing narratives of technology stakeholders' structural experiences is thus becoming a valued practice in contesting AI. However, stories that reflect experiences of racism through technology and in computer science may lack an immediate analysis of how such experiences came about (see for example Ogbonnaya-Ogburu et al., 2020). This is not to say that the stories do not matter or should not be presented. Rather, following Perpich (2010, p. 18), story-telling and autobiography to contest AI should be combined with the immediate historical and political contextualization of experience. Contextualizing experience conveys the idea that experiences are not inevitable but depend on how power is constructed, organized, reproduced, challenged, changed, or abandoned in and through technology and society.

#### 3.2.3 Limitation 3: BF draws on modern Western concepts such as race and gender, thus contesting AI-based services for using related concepts appears inconsistent

Black feminist works have been criticized for overwhelmingly relying on the language and concepts (such as race and gender) that have been established in the course of modernity to subordinate Black women (Puar, 2017, 2012; Jung and Costa Vargas, 2021; McKittrick and Wynter, 2015; McKittrick, 2021). Using those concepts in contesting AI, the critique may become less effective or accidentally reinforce problematic ideas that stem from colonial times of modernity. According to Jerath (2021, p. 32), “modernity refers to social, political, and economic conditions and experiences that result from modernization and capitalism.” Modern experience is shaped by industrialization, technological advancement, enlightenment, colonialism, slavery, the emergence of modern nation states, and the institutionalization of human rights in the USA and Europe as a response to the violent effects of modernity (Jerath, 2021). BF is tied to modernity because it has emerged in response to the brutal subordination of Black women during the transatlantic slave trade and its aftermath. BF has also used the language of rights and justice to fight for the recognition of Black women as subjects to human rights. Puar (2012, p. 54) criticizes that intersectionality draws on the language and concepts of modernity because this reinforces the systems of power that enable the subordination of Black women in the first place:

“many of the cherished categories of the intersectional mantra—originally starting with race, class, gender, now including sexuality, nation, religion, age, and disability—are the products of modernist colonial agendas and regimes of epistemic violence, operative through a Western/Euro-American epistemological formation through which the notion of discrete identity has emerged.”

Puar thus condemns that epistemologies established by Euro-American colonialists are the foundation of Black feminist argumentation against the violence enacted by settler colonialists or their descendants. Puar calls for different epistemologies and to reject subjectivity and identity. Puar proposes queer/assemblage theory as a vantage point from which identities can be theorized as flexible and temporal events that transcend boundaries (e.g., between human-animal or nature-culture, Puar, 2012, p. 58). Queer theory, as a product of post-modern and post-structuralist thought, has made important contributions to challenging established gender identities. Judith Butler has argued that feminism works against itself by remaining within the boundaries of a “woman” category which alleges female unity and naturalizes gender (Jagose, 2007, p. 83). Simply using the term “woman” can reproduce harmful gender roles associated with it. But breaking with woman as a category and building other categories walks into the trap of defining new exclusive boundaries. “Queer” resists any definition and upholds flexibility and fluidity in social relations (Jagose, 2007, p. 98).

With regard to contesting AI, queer theory is crucial to reveal the harm experienced by technology stakeholders who defy established categories. Keyes (2019) condemns the need to identify oneself as female or male in a computerized system to gain access and receive services. But even when one is out and identified as trans or queer, this information may be used for profiling (such as targeted content, advertisement, and surveillance). Keyes (2019) describes data science as a profound threat to queer people because the reduction of complex identities to data points denies queer existence.

There have been different reactions to post-modern and post-structuralist perspectives on gender, race, and Black women's subjectivity. One line of response acknowledges that race and gender are socially conceived as part of modern discourses, but abandoning those constructs in analysis would render their effects (racism, sexism) invisible. This is expressed by Hankerson et al. (2016), when they argue that racial bias in technology has real effects on users. When technology does not work for Black people, they feel excluded, which has consequences for their psychological state. The problem is that diversity is often not considered in the creation of personae or avatars in computer games and thus the White male body may be automatically centered as the primary user of a computer system (Hankerson et al., 2016, p. 478).

Another line of response argues that leveraging problematic constructs in identity politics may be the only way to alleviate the crackdown on Black women's lives. Cooper (2016, p. 395) counters Puar's argument against subject formation by saying that it neglects the struggle of Black women in the American context, where the nation state grants rights to subjects. Only those who can establish themselves as subjects will be able to access legal protection (which, arguably, is difficult enough for Black women). Indeed, identity politics has successfully drawn attention to the discriminatory effects of contemporary AI systems. The activism of computer scientists, authors, and activists Joy Buolamwini and Timnit Gebru, who explicitly promote a Black feminist perspective while also raising awareness about the experiences of trans users of technology, have shaped the regulatory efforts around facial recognition technology (Kantayya, 2020). Buolamwini has engaged the central institutions of the American state by testifying in Congress about racial and gender bias in facial recognition technology (Buolamwini, 2019b). Such an approach formulates Black women technology stakeholders as subjects of rights and accepts the American government as a necessary point of contact in recognizing those rights. While this approach alleviates the worst discrimination, underlying ways of thinking/designing that cause systemic marginalization remain unchallenged.

## 4 Discussion

Black feminism is one of the most sophisticated frameworks for social justice. Working with Black feminist theory to contest AI-based algorithmic decision-making or the design of AI-based systems and their implications for different groups of technology stakeholders has enormous potential. Yet there are limitations to challenging data science or AI-based recommendation, matching, classification, evaluation and so on. This is because most of the critique is constrained by the unequal power relations that are firmly embedded in Western language and methods, including methods of programming, designing, and classifying people into subjects of algorithmic mediation (Bonilla-Silva and Zuberi, 2008). This inspires the consideration of constraints in critical research and design more broadly and how researchers and designers can deal with these constraints. In order to successfully, efficiently and lastingly contest discriminatory AI applications, I suspect that a continuum is necessary: from structural critique that aims at the transformation of AI-provoked injustice to smaller, strategic steps toward betterment. The latter may seem unsatisfying for a critical theory but being patient and carving out opportunities within constraints can gradually improve the lived reality of marginalized technology stakeholders.

How can researchers and designers contest problematic AI use considering the constraints that they face? In the process of developing a plurality of strategies toward AI justice, it is important to recognize and name the constraints that researchers and designers are facing. For instance, one constraint is the silencing and dismissal of critical scholars in mainstream discourses, which may be motivated by racism and misogyny. A strategy of working within this constraint is to wait for changed conditions in public discourse. An example of this is the 2023 rise in concern about large language models and generative AI. Leaders in the technology industry and economy have started to warn of the risk of such technology, e.g., Geoffrey Hinton and Joseph Stiglitz (Taylor and Hern, 2023; Bushwick, 2023). Black women had warned much earlier about the issue and have been discredited for naming the dangers of “stochastic parrots” (which is the title of the famous text that played a role in the firing of Black AI ethicist Timnit Gebru from Google, see Bender et al., 2021; Turner et al., 2021). However, the fact that, in 2023, AI and economic leaders publicly voiced concern can be an opportunity for scholars working with critical approaches to reiterate their arguments and move their critique of unregulated AI into the mainstream.

Another constraint is the economic context within which critical research on AI is produced, as academic and industrial spaces are shaped by neoliberal capitalism. Given this constraint, alternative business models and infrastructures for design have been proposed (cf. Scholz and Schneider, 2017). Smyth and Dimond (2014, p. 70) propose cooperatives instead of companies to make the work environment where design is facilitated anti-oppressive so that the design can be anti-oppressive as well. Smyth and Dimond (2014, p. 71) write:

“Worker co-ops tend to create long-term, stable jobs and a concern for community benefit. Many espouse a ‘multiple bottom line,' wherein the business's objectives are not limited to financial returns and include other values such as environmental sustainability, community impact, and worker happiness.”

Examples of existing worker cooperatives in the technology sector are Sassafras Tech Collective^3^ and Research Action Design.^4^ In the context of online platforms, Poster (2019, p. 163) suggests “platform cooperativism” as a new model for more just online environments. They propose to look to the history of African-American cooperatives for models of platform infrastructures where ownership and governance is in users' hands (e.g., inspiration can be found in W.E.B DuBois'1907 text “Economic Cooperation among Negro Americans”). While these initiatives are promising, the reality is that most research and design takes place in contexts where neoliberal capitalism dictates design decisions.

This is not only true for big technology companies that demand the alignment of their products with company interests. Funding from governments, intergovernmental organizations (such as the European Union) and foundations can similarly put constraints on researchers and designers, who may further engage in self-censorship to avoid agitating potential funders (Wolf et al., 2022, p. 444). There is also a (perceived) need to scale new AI-based products or processes as a high adoption rate is a measure for success (Wolf et al., 2022, p. 444). Additionally, there is time pressure. Research projects are usually funded for a few years (if at all), pressuring researchers to go in and out of communities with little concern for their long-term development. Time is entangled with the “publish or perish culture” in academia where a researcher is incentivized to produce numerous written outputs in a short amount of time (cf. Erete et al., 2023, p. 26f). Little or no funding is offered to community members who participate in the research or design project (Tran O'Leary et al., 2019, p. 8).

In light of the neoliberal framework for contesting harmful AI, researchers may focus on small-scale, local, and community-driven activities to consider how power relations materialize in a specific location/history. Such local design processes and the resulting technologies thrive not because of scale but because they build on the needs and assets of a particular community. Another possibility to engage in critical work on AI is to reflect the meaning of alleged “diversity” concepts that are widely perceived as self-evident in data science (e.g., race, gender). Reflection (which comes down to theoretical work) may seem unsatisfying as it creates neither immediate change nor a product or a solution. However, changing the minds (or at least destabilizing established views) is quite radical in the literal meaning of the word as it goes to the roots of the dominant value and knowledge systems which shape socio-technical relations. Finally, working within but also stretching the boundaries of constraints can best be done in cooperation because this gives more weight to the work of researchers and designers using critical approaches. For example, there can be cooperation between different feminist movements with a focus on “designing across difference” and uplifting different critical approaches at the same time.

## 5 Conclusion

This paper has shown that a critical social theory like American Black feminism has tremendous potential but also limitations to contest harmful AI applications and their implications. It has highlighted that there are constraints on the critical scholarship and design of AI-based technologies, which come back to unequal power relations in society, between individuals and institutions, and biased knowledge systems deeply engrained in scholarly consciousness. Still, lots can be done within those constraints to challenge discriminatory AI-based systems and harmful methods of classification, sorting, and profiling via algorithmic mediation. This includes the strategic use of established (harmful) language and methods as well as strategic essentialization to get the attention of the mainstream discourse but then transforming concepts and ideas that have a history of colonization and exclusion. Further promising approaches include (a) critical reflection of notions of diversity or difference that are increasingly embedded in AI but risk reinforcing societal inequalities, (b) historical contextualization of AI applications and their effects (e.g., the case of the “Whiteness” of AI-systems' humanoid presentations), as well as (c) intersectional-type analysis of the works of power in and through AI, including the effects of race and gender regimes in combination with less recognized power systems like language, religion, and wealth. This paper hopefully has inspired the application and testing of the above strategies and motivated the use of American Black feminism in studies of AI more broadly. Increasing the number of critical studies on AI systems and their effects on diverse groups of technology stakeholders helps not only make these (computer) systems better but also learn more about the practice of contesting powerful socio-technical systems beyond AI.

## Data Availability

The original contributions presented in the study are included in the article/supplementary material, further inquiries can be directed to the corresponding author.

## Footnotes

^1^I am myself a White European woman who has applied American Black feminist traditions to the study and design of “diversity-aware technology.” While I acknowledge the limitations that my perspective brings to applying Black feminism, I believe that, if the theory is well-suited to analyze the topic at hand, a refusal to apply Black feminism due to the Whiteness of the scholar would further marginalize it. Promoting Black feminist tools of analysis from diverse positionalities may all in all contribute to better critical AI research and development.

^2^Power systems that have received less attention in Black feminism include religion (the norm is Christianity), language (the norm is English) and wealth (the norm is wealth accumulation as opposed to redistribution). I will come back to these power systems later in this paper.

^3^ http://www.sassafras.coop/

^4^ https://rad.cat

## References

[B1] Ahmed N. Tasmin M. Ibrahim S. M. N. (2022). Technology for empowerment: context of urban Afghan women. Technol. Soc. 70, 1–6. 10.1016/j.techsoc.2022.10205839021531

[B2] Alden C. (2003). Let them eat cyberspace: Africa, the G8 and the digital divide. Millennium 32, 457–476. 10.1177/03058298030320030601

[B3] Bender E. M. Gebru T. McMillan-Major A. Shmitchell S. (2021). “On the dangers of stochastic parrots: can language models be too big?,” in FAccT '21: Proceedings of the 2021 ACM Conference on Fairness, Accountability, and Transparency (New York, NY: ACM), 610–623. 10.1145/3442188.3445922

[B4] Benjamin R. (ed.). (2019a). Captivating Technology: Race, Carceral Technoscience, and Liberatory Imagination in Everyday Life. Durham, NC: Duke University Press. 10.1215/9781478004493

[B5] Benjamin R. (2019b). Race After Technology: Abolitionist Tools for the New Jim Code. Cambridge; Medford, MA: Polity Press.

[B6] Bonilla-Silva E. Zuberi T. (2008). White Logic, White Methods: Racism and Methodology. Lanham, MD: Rowman and Littlefield Publishers.

[B7] Brah A. Phoenix A. (2004). Ain't I a woman? Revisiting intersectionality. J. Int. Wome's Stud. 5, 75–86.

[B8] Browne J. Stephen Cave E. D. McInerney K. (eds.). (2023). Feminist AI: Critical Perspectives on Data, Algorithms and Intelligent Machines. Oxford: Oxford University Press. 10.1093/oso/9780192889898.001.0001

[B10] Buolamwini J. (2019a). Response: Racial and Gender Bias in Amazon Rekognition - Commercial AI System for Analyzing Faces. Available online at: https://medium.com/@Joy.Buolamwini/response-racial-and-gender-bias-in-amazon-rekognition-commercial-ai-system-for-analyzing-faces-a289222eeced (Accessed February 09, 2019).

[B11] Buolamwini J. (2019b). Artificial Intelligence: Societal and Ethical Implications: Testimony at United States House Committee on Science, Space and Technology. Unpublished manuscript. Available online at: https://www.congress.gov/event/116th-congress/house-event/109688 (Accessed September 09, 2023).

[B12] Buolamwini J. (2023). Unmasking AI: My Mission to Protect What Is Human in a World of Machines. 1st Edn. New York, NY: Random House.

[B13] Buolamwini J. Gebru T. (2018). “Gender shades: intersectional accuracy disparities in commercial gender classification,” in Proceedings of Machine Learning Research, Vol. 81, 1–15. Available online at: http://proceedings.mlr.press/v81/buolamwini18a/buolamwini18a.pdf (Accessed August 18, 2018).

[B14] Bushwick S. (2023). Unregulated AI Will Worsen Inequality, Warns Nobel-Winning Economist Joseph Stiglitz. Scientific American, August 1. Available online at: https://www.scientificamerican.com/article/unregulated-ai-will-worsen-inequality-warns-nobel-winning-economist-joseph-stiglitz/ (Accessed September 22, 2023).

[B15] Cave S. Dihal K. (2020). The whiteness of AI. Philos. Technol. 33, 685–703. 10.1007/s13347-020-00415-6

[B16] Chin R. (2017). The Crisis of Multiculturalism in Europe: A History. Princeton: Princeton University Press. 10.1515/9781400884902

[B17] Coleman D. (2019). Digital colonialism: the 21st century scramble for Africa through the extraction and control of user data and the limitations of data protection laws. Michigan J. Race Law 24, 417–439. 10.36643/mjrl.24.2.digital

[B18] Collins P. H. (2000a). Black Feminist Thought: Knowledge, Consciousness, and the Politics of Empowerment. Perspectives on gender, 2nd Edn. New York, NY; London: Routledge.

[B19] Collins P. H. (2000b). Gender, black feminism, and black political economy. Ann. Am. Acad. Polit. Soc. Sci. 568, 41–53. 10.1177/000271620056800100512288804

[B20] Collins P. H. (2015). Intersectionality's definitional dilemmas. Annu. Revi. Sociol. 41, 1–20. 10.1146/annurev-soc-073014-112142

[B21] Collins P. H. Bilge S. (2016). Intersectionality: Key Concepts. Cambridge; Malden, MA: Polity Press.

[B22] Cooper B. C. (2016). “Intersectionality,” in The Oxford Handbook of Feminist Theory, eds. J. Lisa Disch and M. E. Hawkesworth (Oxford; New York, NY: Oxford University Press), 385–406. 10.1093/oxfordhb/9780199328581.013.20

[B23] Costanza-Chock S. (2020). Design Justice: Community-Led Practices to Build the Worlds We Need. Information Policy. Cambridge: The MIT Press. 10.7551/mitpress/12255.001.0001

[B24] Crenshaw K. (1989). Demarginalizing the Intersection of Race and Sex: A Black Feminist Critique of Antidiscrimination Doctrine, Feminist Theory and Antiracist Politics. University of Chicago Legal Forum, 139–167. Available online at: https://chicagounbound.uchicago.edu/cgi/viewcontent.cgi?article=1052&context=uclf (Accessed June 02, 2020).

[B25] Crenshaw K. (1991). Mapping the margins: intersectionality, identity politics, and violence against women of color. Stanford Law Rev. 43, 241–1299. 10.2307/1229039

[B26] Crenshaw K. (1995). “Mapping the margins: intersectionality, identity politics, and violence against women of color,” in Critical Race Theory: The Key Writings That Formed the Movement, eds. K. Crenshaw, N. Gotanda, G. Peller, and K. Thomas (New York, NY: New Press), 357–383.

[B27] Criado Perez C. (2019). Invisible Women. Data Bias in a World Designed for Men. New York, NY: Abrams.

[B28] Daniels J. (2015). My brain database doesn't see skin color: color-blind racism in the technology industry and in theorizing the web. Am. Behav. Scientist 59, 1377–1393. 10.1177/0002764215578728

[B29] Day K. (2016). Religious Resistance to Neoliberalism: Womanist and Black Feminist Perspectives. Basingstoke; Hampshire; New York, NY: Palgrave Macmillan. 10.1007/978-1-137-56943-1

[B30] Dhamoon R. (2015). A Feminist Approach to Decolonizing Anti-Racism: Rethinking Transnationalism, Intersectionality, and Settler Colonialism, 20–37. Available online at: https://feralfeminisms.com/wp-content/uploads/2015/12/ff_A-Feminist-Approach-to-Decolonizing-Anti-Racism_issue4.pdf (Accessed March 11, 2020).

[B31] D'Ignazio C. Klein L. F. (2020). Data Feminism. Cambridge, MA: The MIT Press. 10.7551/mitpress/11805.001.0001

[B32] Ekstrand M. D. Tian M. Imran Kazi M. R Mehrpouyan H. Kluver D. (2018). “Exploring author gender in book rating and recommendation,” in Proceedings of the 12th ACM Conference on Recommender Systems, ed. S. Pera (New York, NY: ACM), 242–250. 10.1145/3240323.324037338864075

[B33] Emejulu A. Sobande F. (2019). To Exist Is to Resist: Black Feminism in Europe. 1st Edn. London: Pluto Press. 10.2307/j.ctvg8p6cc

[B34] Erete S. Israni A. Dillahunt T. (2018). An intersectional approach to designing in the margins. Interactions 25, 66–69. 10.1145/3194349

[B35] Erete S. Rankin Y A. Thomas J. O. (2023). A method to the madness: applying an intersectional analysis of structural oppression and power in HCI and design. ACM Trans. Comput. Hum. Interact. 30, 1–45. 10.1145/3507695

[B36] Fabris A. Baranowska N. Dennis M. J Graus DF. Hacker P. Saldivar J.. (2025). Fairness and bias in algorithmic hiring: a multidisciplinary survey. ACM Trans. Intell. Syst. Technol. 16, 1–54. 10.1145/3696457

[B37] Feagin J. R. (2006). Systemic Racism: A Theory of Oppression. New York, NY: Routledge.

[B38] Garske P. (2013). “Intersektionalität Als herrschaftskritik? Die kategorie ‘klasse' und das gesellschaftskritische potenzial der intersektionalitäts-debatte,” in Intersectionality Und Kritik, eds. J. M. Kallenberg and J. M. Müller (Wiesbaden: Springer Fachmedien Wiesbaden), 245–263. 10.1007/978-3-531-93168-5_12

[B39] Guy-Sheftall B. (ed.). (1996). Words of Fire: An Anthology of African-American Feminist Thought. 2nd Edn. New York, NY: The New Press.

[B40] Hacker P. (2025). Oxford Intersections: AI in Society. Oxford: Oxford University Press. 10.1093/9780198945215.001.0001

[B41] Hampton L. M. (2021). “Black feminist musings on algorithmic oppression,” in Proceedings of the 2021 ACM Conference on Fairness, Accountability, and Transparency (New York, NY: ACM), 1–12.

[B42] Hanemaayer A. (ed.). (2022). Artificial Intelligence and Its Discontents. Social and Cultural Studies of Robots and AI. Cham: Springer International Publishing. 10.1007/978-3-030-88615-8

[B43] Hankerson D. Marshall A R. Booker J. El Mimouni H. Walker I. Rode J A. (2016). “Does technology have race?,” in Proceedings of the 2016 *CHI Conference on Human Factors in Computing Systems*, eds. J. Kaye, A. Druin, C. Lampe, D. Morris, and Hourcade J. P (New York, NY: ACM), 473–486. 10.1145/2851581.2892578

[B44] Hannák A. Wagner C. Garcia D. Mislove A. Strohmaier M. Wilson C. (2017). “Bias in online freelance marketplaces,” in Proceedings of the 2017 ACM International Conference on Computer-Supported Cooperative Work and Social Computing, eds. C. P. Lee, S. Poltrock, L. Barkhuus, M. Borges and W. Kellogg (New York, NY: ACM), 1914–1933. 10.1145/2998181.2998327

[B45] Heesen J. Reinhardt K. Schelenz L. (2021). “diskriminierung durch algorithmen vermeiden: analysen und instrumente für eine demokratische digitale gesellschaft,” in Diskriminierung Und Antidiskriminierung: Beiträge Aus Wissenschaft Und Praxis, 1st Edn, ed. G. Bauer, M. Kechaja, S. Engelmann, and L. Haug,” (Bielefeld: Transcript Verlag). 10.1515/9783839450819-008

[B46] Himmelsbach J. Schwarz S. Gerdenitsch C. Wais-Zechmann B. Bobeth J. Tscheligi M. (2019). “Do we care about diversity in human computer interaction: a comprehensive content analysis on diversity dimensions in research,” in CHI '19: Proceedings of the 2019 CHI Conference on Human Factors in Computing Systems (Glasgow: ACM), 1–16. 10.1145/3290605.3300720

[B47] Hoffmann A. L. (2020). Terms of inclusion: data, discourse, violence. N. Media Soc. 23, 3539–3556. 10.1177/146144482095872516499246

[B48] Hooks B. (2015a). Ain't I a Woman: Black Women and Feminism, 2nd Edn. New York, NY: Routledge.

[B49] Hooks B. (2015b). Black Looks: Race and Representation, New Edn. New York, NY: Routledge. 10.4324/9781315743226

[B50] Howard A. Borenstein J. (2018). The ugly truth about ourselves and our robot creations: the problem of bias and social inequity. Sci. Eng. Ethics 24, 1521–1536. 10.1007/s11948-017-9975-2 28936795

[B51] Jagose A. (2007). Queer Theory: An Introduction. Reprinted. New York, NY: New York University Press.

[B52] Jerath K. S. (2021). Science, Technology and Modernity: An Interdisciplinary Approach. 1st Edn. Cham: Springer International Publishing. 10.1007/978-3-030-80465-7

[B53] Jung M.-K. Costa Vargas J. H. (2021). Antiblackness. Durham: Duke University Press. 10.1215/9781478013167

[B54] Kantayya S. (2020). Coded Bias. Documentary.

[B55] Kärkkäinen K. Joo J. (2019). FairFace: Face Attribute Dataset for Balanced Race, Gender, and Age. Available online at: https://arxiv.org/pdf/1908.04913 (Accessed September 21, 2023).

[B56] Kelly M. (2023). Wealth Supremacy: How the Extractive Economy and the Biased Rules of Capitalism Drive Today's Crises. Oakland, CA: Berrett-Koehler Publishers.

[B57] Keyes O. (2019). Counting the Countless: Why Data Science Is a Profound Threat for Queer People. Real Life Magazine. Available online at: https://reallifemag.com/counting-the-countless/ (Accessed July 18, 2019).

[B58] Khan F. M. (2018). Access and beyond: an intersectional approach to women's everyday experiences with ICTs. Netw. Knowl. J. MeCCSA Postgraduate Netw. 11, 5–20. 10.31165/nk.2018.112.53337162741

[B59] Kirk-Duggan C. (2014). Womanist Theology as a Corrective to African American Theology. In The Oxford Handbook of African American Theology, edited by Anthony Pinn B, Katie G. Cannon, CO: Oxford University Press.

[B60] Kumar N. Karusala N. (2019). Intersectional computing. Interactions 26, 50–54. 10.1145/3305360

[B61] Liang C. A. Munson S A. Kientz J. A. (2021). Embracing four tensions in human-computer interaction research with marginalized people. ACM Trans. Comput. Hum. Interact. 28, 1–47. 10.1145/3443686

[B62] Lorde A. (1980). Age, Race, Class and Sex: Women Redefining Difference: Paper Delivered at the Copeland Colloquium, Amerst College. Available online at: https://www.colorado.edu/odece/sites/default/files/attached-files/rba09-sb4converted_8.pdf (Accessed May 13, 2020).

[B63] Marshall B. H. (2023). Data Conscience: Algorithmic Siege on Our Humanity. With the assistance of T. Gebru. Hoboken, NJ: Wiley. 10.1002/9781394320721

[B64] McKittrick K. (2021). Dear Science and Other Stories. Errantries. Durham, NC: Duke University Press. 10.1215/9781478012573

[B65] McKittrick K. Wynter S. (eds.). (2015). Sylvia *Wynter: On Being Human as Praxis*. Durham, NC: Duke University Press. 10.1515/9780822375852

[B66] McNee S. M. Riedl J. Konstan J. A. (2006). “Being accurate is not enough,” in CHI '06 Extended Abstracts on Human Factors in Computing Systems, eds. G. Olson and R. Jeffries (New York, NY: ACM), 1097–1101. 10.1145/1125451.1125659

[B67] Mirza H. S. (2009). Plotting a history: black and postcolonial feminisms in ‘new times'. Race Ethnicity Educ. 12, 1–10. 10.1080/13613320802650899

[B68] Mustafa M. Lazem S. Alabdulqader A. (2020). “IslamicHCI: designing with and within muslim populations,” in Extended Abstracts of the 2020 CHI Conference on Human Factors in Computing Systems, eds. R. Bernhaupt, F. Mueller, D. Verweij, J. Andres, J. McGrenere, A. Cockburn, et al. (New York, NY: ACM), 1–8. 10.1145/3334480.3375151

[B69] Nash J. C. (2008). Re-thinking intersectionality. Fem. Rev. 89, 1–15. 10.1057/fr.2008.4

[B70] Noble S. U. (2016). A Future for Intersectional Black Feminist Technology Studies. The Scholar and Feminist Online. Available online at: http://sfonline.barnard.edu/traversing-technologies/safiya-umoja-noble-a-future-for-intersectional-black-feminist-technology-studies/ (Accessed June 27, 2020).

[B71] Noble S. U. (2018). Algorithms of Oppression: How Search Engines Reinforce Racism. New York, NY: New York University Press. 10.18574/nyu/9781479833641.001.0001 34709921

[B72] Norwood C. (2013). Perspective in Africana feminism; exploring expressions of black feminism/womanism in the African Diaspora. Sociol. Compass 7, 225–236. 10.1111/soc4.12025

[B73] Ogbonnaya-Ogburu I. F. Finda I. Smith AD R. To A. Toyama K. (2020). “Critical race theory for HCI,” in Proceedings of the 2020 CHI Conference on Human Factors in Computing Systems, eds R. Bernhaupt, F. Mueller, D. Verweij, J. Andres, J. McGrenere, A. Cockburn, et al. (New York, NY: ACM), 1–16. 10.1145/3313831.3376392

[B74] Oneto L. Chiappa S. (2020). “Fairness in machine learning,” in Recent Trends in Learning from Data. Studies in Computational Intelligence, Vol. 896, eds. L. Oneto, N. Navarin, A. Sperduti, and D. Anguita (Cham: Springer International Publishing), 155–196. 10.1007/978-3-030-43883-8

[B75] Perpich D. (2010). “Black feminism, poststructuralism, and the contested character of experience,” in Convergences: Black Feminism and Continental Philosophy. SUNY Series in Gender Theory, eds. L. Donna-Dale Marcano, K. T. Gines, and M. Del Guadalupe Davidson (Albany, NY: SUNY Press), 13–34.

[B76] Piela A. (2012). Muslim Women Online: Faith and Identity in Virtual Space. Routledge Islamic Studies Series. New York, NY: Routledge.

[B77] Poster W. (2019). “Racialized surveillance in the digital service industry,” in Captivating Technology: Race, Carceral Technoscience, and Liberatory Imagination in Everyday Life, ed. R. Benjamin (Durham, NC: Duke University Press), 133–169. 10.1215/9781478004493-009

[B78] Puar J. K. (2012). I would rather be a cyborg than a goddess: becoming-intersectional in assemblage theory. philoSOPHIA 2, 49–66. 10.1353/phi.2012.a48662134409987

[B79] Puar J. K. (2017). Terrorist Assemblages: Homonationalism in Queer Times. Next Wave. Durham, NC, London: Duke University Press. 10.1215/9780822371755

[B80] Rankin Y. A. Thomas J. O. Erete S. (2021). “Real talk: saturated sites of violence in CS education,” in Proceedings of the 52nd ACM Technical Symposium on Computer Science Education, ed. M. Sherriff (New York, NY: ACM), 802–808.

[B81] Reddy M. T. (1998). Invisibility/hypervisibility: the paradox of normative whiteness. SPECIAL ISSUE: how could you not hear it? Writings on race · color · whiteness. Transformations 9, 55–64.

[B82] Rhee J. (2018). The Robotic Imaginary: The Human and the Price of Dehumanized Labor. Minneapolis, MN: University of Minnesota Press. 10.5749/j.ctv62hh4x

[B83] Robinson B. G. (2019). Racialization and modern religion: Sylvia Wynter, black feminist theory, and critical genealogies of religion. Crit. Res. Relig. 7, 257–274. 10.1177/2050303219848065

[B84] Rodriguez C. R. Tsikata D. Ampofo A. A. (eds.). (2016). Transatlantic Feminisms: Women and Gender Studies in Africa and the Diaspora. With the assistance of Y. Bass, A. L. Bolles, M. Celeste, B. A. Ezati, M. Mbilinyi, J. L. McBrien, et al. (Lanham, MD: Lexington Books). 10.5040/9781978740006

[B85] Rusert B. (2017). Fugitive Science: Empiricism and Freedom in Early African American Culture. America and the Long 19th Century. New York, NY: New York University Press.

[B86] Sabie D. Ahmed S. I. (2019). “Moving into a technology land,” in Proceedings of the 2nd ACM SIGCAS Conference on Computing and Sustainable Societies, eds. J. Chen, J. Mankoff, and C. Gomes (New York, NY: ACM), 218–233. 10.1145/3314344.3332481

[B87] Salem S. Thompson V. (2016). Old racisms, new masks: on the continuing discontinuities of racism and the erasure of race in European contexts. Nineteen Sixty Nine 3, 1–23. Available online at: https://escholarship.org/uc/item/98p8q169

[B88] Salinas A. Haim A. Nyarko J. (2024). What's in a Name? Auditing Large Language Models for Race and Gender Bias. http://arxiv.org/pdf/2402.14875v3 (Accessed January 13, 2025).

[B89] Schelenz L. (2022a). “Artificial intelligence between oppression and resistance: black feminist perspectives on emerging technologies,” in Artificial Intelligence and Its Discontents. Social and Cultural Studies of Robots and AI, ed. A. Hanemaayer (Cham: Springer International Publishing), 225–249. 10.1007/978-3-030-88615-8_11

[B90] Schelenz L. (2022b). Diversity concepts in computer science and technology development: a critique. Sc. Technol. Hum. Values 48, 1054–1079. 10.1177/01622439221122549

[B91] Schelenz L. (2023a). Diversity and social justice in technology design: reflections on diversity-aware technology. Int. J. Crit. Diver. Stud. 5. 10.13169/intecritdivestud.5.2.0033

[B92] Schelenz L. (2023b). Technology, power, and social inclusion: afghan refugee women's interaction with ICT in Germany. Int. J. Inform. Diver. Inclusion 7, 1–31. 10.33137/ijidi.v7i3/4.40292

[B93] Schelenz L. Reinhardt K. Gjuraij D. (2019). Developing a Conceptual and Ethical Framework of Diversity: Deliverable 9.1 for the Project WeNet- the Internet of Us. (Unpublished manuscript) Available online at: https://internetofus.eu/wp-content/uploads/sites/38/2019/07/D9.1-EKUT-Ethical-Framework-final.pdf (Accessed May 12, 2023).

[B94] Scholz T. Schneider N. (2017). Ours to Hack and to Own: The Rise of Platform Cooperativism, a New Vision for the Future of Work and a Fairer Internet. New York, NY: OR Books. 10.2307/j.ctv62hfq7

[B95] Scott J. W. (1992). “Experience,” in Feminists Theorize the Political, eds. J. Butler and J. W. Scott (New York, NY: Routledge), 22–40.

[B96] Shahalimi N. (ed.). (2022). Wir Sind Noch Da! Mutige Frauen Aus Afghanistan, 2nd Edn. München: Elisabeth Sandmann Verlag.

[B97] Smyth T. Dimond J. (2014). Anti-oppressive design. Interactions 21, 68–71. 10.1145/2668969

[B98] Sonboli N. Burke R. Liu Z. Mansoury M. (2020). “Fairness-aware recommendation with librec-auto,” in Fourteenth ACM Conference on Recommender Systems, ed. N.A. (New York, NY: ACM), 594–596. 10.1145/3383313.3411525

[B99] Subramaniam B. (2014). Ghost Stories for Darwin: The Science of Variation and the Politics of Diversity. Urbana, IL: University of Illinois Press. 10.5406/illinois/9780252038655.001.0001

[B100] Taylor J. Hern A. (2023). ‘*Godfather of AI' Geoffrey Hinton Quits Google and Warns over Dangers of Misinformation*. Available online at:https://www.theguardian.com/technology/2023/may/02/geoffrey-hinton-godfather-of-ai-quits-google-warns-dangers-of-machine-learning (Accessed September 22, 2023).

[B101] Thomas J. O. Joseph N. Williams A. Crum C. Burge J. (2018). “Speaking truth to power: exploring the intersectional experiences of black women in computing,” in 2018 Research on Equity and Sustained Participation in Engineering, Computing, Technology (RESPECT) (Baltimore, MD: IEEE), 1–8. 10.1109/RESPECT.2018.8491718

[B102] Tran O'Leary J. Zewde S. Mankoff J. Rosner D K. (2019). Who “Gets to future? Race, representation, and design methods in Africa town,” in *CHI '19: Proceedings of the 2019 CHI Conference on Human Factors in Computing System* (Glasgow: ACM), 1–13. 10.1145/3290605.3300791

[B103] Turman E. M. (2016). Toward a Womanist Ethic of Incarnation: Black Bodies, the Black Church, and the Council of Chalcedon. New York, NY: Palgrave Macmillan.

[B104] Turner K. Wood D. D'Ignazio C. (2021). The Abuse and Misogynoir Playbook. originally appeared in the January Issue of the Quarterly State of AI Ethics Report published by the Montreal AI Ethics Institute. Available online at: https://www.media.mit.edu/publications/abuse-and-misogynoir-playbook/ (Accessed Juyl 2, 2021).

[B105] Twine F. W. (2018). Technology's invisible women: black geek girls in silicon valley and the failure of diversity initiatives. Int. J. Crit. Diver. Stud. 1, 58–79. 10.13169/intecritdivestud.1.1.0058

[B106] Wachter-Boettcher S. (2017). Technically Wrong: Sexist Apps, Biased Algorithms, and Other Threats of Toxic Tech. New York, NY: W.W. Norton and Company.

[B107] Warschauer M. (2004). Technology and Social Inclusion: Rethinking the Digital Divide. Cambridge, MA: The MIT Press. 10.7551/mitpress/6699.001.0001

[B108] Weber B. M. (2015). Gender, race, religion, faith? Rethinking intersectionality in german feminisms. Eur. J. Womens Stud. 22, 22–36. 10.1177/1350506814552084

[B109] Weibert A. Krüger M. Aal K. Salehee S. S. Khatib R. Randall D.. (2019). Finding language classes: designing a digital language wizard with refugees and migrants. Proc. ACM Hum.Comput. Interact. 3, 1–23. 10.1145/335921834322658

[B110] Windle J. A. de Jesus D. Bartlett L. (2020). The Dynamics of Language and Inequality in Education. Bristol: Multilingual Matters. 10.21832/WINDLE6942

[B111] Wolf C. T. Asad M. Dombrowski L. S. (2022). “Designing within capitalism,” in Designing Interactive Systems Conference, eds. Mueller, F., S. Greuter, R. A. Khot, P. Sweetser, and M. Obrist (New York, NY: ACM), 439–453. 10.1145/3532106.3533559

[B112] Wong-Villacres M. Kumar A. Vishwanath A. Karusala N. DiSalvo B. Kumar N. (2018). “Designing for intersections,” in Proceedings of the 2018 Designing Interactive Systems Conference, eds. I. Koskinen, Y. Lim, T. Cerratto-Pargman, K. Chow, and W. Odom (New York, NY: ACM), 45–58. 10.1145/3196709.3196794

[B113] Wynter S. (2015). “On how we mistook the map for the territory, and re-imprisoned ourselves in our unbearable wrongness of being, of Désêtre: black studies toward the human project,” in Not Only the Master's Tools: African American Studies in Theory and Practice, eds. L. R. Gordon and J. A. Gordon (New York, NY: Routledge).

[B114] Yuval-Davis N. (2006). Intersectionality and feminist politics. Eur. J. Womens Stud. 13, 193–209. 10.1177/1350506806065752

[B115] Zou J. Schiebinger L. (2018). AI can be sexist and racist - it's time to make it fair. Nature 559, 324–326. 10.1038/d41586-018-05707-8 30018439

[B116] Zuboff S. (2019). The Age of Surveillance Capitalism: The Fight for a Human Future at the New Frontier of Power. London: Profile Books.

